# Role of Dexmedetomidine in Early POCD in Patients Undergoing Thoracic Surgery

**DOI:** 10.1155/2021/8652028

**Published:** 2021-11-23

**Authors:** Jiao Ran, Xiao Bai, Rurong Wang, Xuehan Li

**Affiliations:** Department of Anesthesiology, West China Hospital, Sichuan University & the Research Units of West China (2018RU012), Chinese Academy of Medical Sciences, China

## Abstract

**Objective:**

To evaluate whether a low-dose perioperative infusion of Dex reduces early POCD.

**Design:**

This study was a double-blind, randomized, placebo-controlled trial that randomly assigned patients to Dex or saline placebo infused during surgery and patient-controlled intravenous analgesia (PCIA) infusion. Patients were assessed for postoperative cognitive decline. *Interventions*. Dex was infused at a loading dose of 0.5 *μ*g/kg intravenously (15 min after entering the operation room) followed by a continuous infusion at a rate of 0.5 *μ*g/kg/h until one-lung ventilation or artificial pneumothorax ended. Patients in the Dex group received regular PCIA pump with additional dose of Dex (200 *μ*g).

**Results:**

In total, 126 patients were randomized, and 102 patients were involved in the result analysis. The incidence of POCD was 36.54% (19/52) in the Dex group and 32.00% (16/50) in the normal saline (NS) group, with no statistic difference. No significant difference was observed between the two groups in terms of Telephone Interview for Cognitive Status-Modified (TICS-m) scores at different times. However, the TICS-m score at 7 days after surgery was significantly lower than that at 30 days in 102 patients (32.93 ± 0.42 vs. 33.92 ± 0.47, *P* = 0.03). The visual analogue scale scores in the Dex group were significantly lower than those in the NS group 1 day postoperation at rest and activity (2.00 [1.00–3.00] vs. 3.00 [2.00–4.00], *P* < 0.01; 4.00 [3.00–5.00] vs. 5.00 [4.00–6.00], *P* < 0.05, respectively). Patients receiving Dex or NS had no statistical difference in activities of daily living (ADLs) scores at 7 and 30 days after surgery, but the ADL score at 30 days after surgery showed a significant reduction compared with that at 7 days (*P* < 0.01). Patients in the Dex group had a shorter hospital length of stay (15.26 ± 3.77 vs. 17.69 ± 5.09, *P* = 0.02) and less expenses (52458.71 ± 10649.30 vs. 57269.03 ± 9269.98, *P* = 0.04) than those in the NS group.

**Conclusions:**

Low-dose Dex in the perioperative period did not reduce the incidence of early POCD in thoracic surgery. However, it relieved postoperative pain, decreased the hospitalization expenses, and shortened the length of stay.

## 1. Introduction

Since the emergence of neuropsychological tests to objectively assess cognitive changes after cardiac surgery in the 1980s, postoperative neurocognitive disorder associated with anesthesia and surgery has become the concern of anesthesiologists [1]. The incidence of postoperative cognitive dysfunction (POCD) ranges from 6% to 53% [2, 3], varying with age, education level, comorbidity, and surgery. Moreover, inconsistent methodologies may result in the variation of its incidence [4, 5].

The underlying pathogenesis of POCD is the neuroinflammation and oxidative stress from anesthesia and surgery [6, 7]. Surgical trauma provokes the release of proinflammatory cytokines. Neuroinflammation may impair neural cells and increase the peripheral blood levels of neuron damage-associated biochemical markers.

Thoracic surgery is accompanied with one-lung ventilation (OLV) with double-lumen endotracheal tube or two-lung ventilation with CO_2_ artificial pneumothorax. No matter which ventilation method is adopted, ventilator-induced lung injury is clinically significant, as it induces direct lung injury and systemic inflammation [8, 9]. In addition, an imbalanced ventilation/perfusion ratio can directly decrease the cerebral oxygen saturation and harm the cognition-related region [10].

On the basis of the neuroinflammatory hypothesis involved in POCD, anti-inflammatory and antioxidative stress strategies are potential treatments for patients undergoing thoracic surgery [6]. Dexmedetomidine (Dex), a highly selective *α*2-adrenergic receptor agonist, is widely used for sedation and analgesia. Dex relieves stress from surgery and anesthesia via the noradrenergic system [11]. In animal and human studies, Dex can inhibit the release of proinflammatory cytokines [7]. Wu et al. found that Dex could attenuate the inflammatory response in thoracic surgery [12]. Pavone et al. thought that higher infusion rate and longer duration of drug administration may be more effective in improving postoperative cognitive function [13]. Su et al. indicated that prophylactic low-dose Dex infusion (0.1 *μ*g/kg/h, from intensive care unit admission on the day of surgery until 08:00 h on postoperative day 1) could reduce the prevalence of postoperative delirium [14]. Nevertheless, other studies held the view that Dex did not prevent postoperative delirium [15]. Considering that Dex has a dose-dependent effect on POCD, we hypothesized that Dex administration during operation and patient-controlled intravenous analgesia (PCIA) with Dex could reduce the incidence of POCD in patients undergoing thoracic surgery.

## 2. Methods

The prospective, randomized, double-blind, and controlled study protocol was approved by the Ethics Committee of West China Hospital, Sichuan University [2019 (2019042508)] and registered in the Chinese Clinical Trials Registry (ChiCRT-IOR-16008837). We obtained written informed consent from all participants.

### 2.1. Patients and Study Design

We recruited 126 patients aged 45 years or older who underwent elective thoracic surgery with American Society of Anesthesiologists class I–III from July 2019 to December 2019. Twelve nonoperative patients were included to evaluate the learning effects by repeated test of Repeatable Battery for the Assessment of Neuropsychological Status (RBANS). Patients would be excluded if they met any of the exclusion criteria: Telephone Interview for Cognitive Status-Modified (TICS-m) score less than 28, a history of neurological disease, psychiatric and antidepression drugs, education level under primary school, inability to communicate because of dysaudia and vision disorder, serious hepatic dysfunction (Child–Pugh class C) and renal dysfunction (undergoing dialysis), sick sinus syndrome, severe sinus bradycardia (<50 beats per min), or second- or third-degree atrioventricular block without pacemaker. The exit criteria were anesthesia duration less than 2 h, inability to accomplish the cognitive assessment, and reoperation after surgery.

Patients were randomized into two groups: (1) Dex group, with a bolus of 0.5 *μ*g/kg of Dex (Jiangsu Hengrui Medicine Co., Ltd., Jiangsu, China; specification 2 mL: 200 *μ*g) (15 min after entering the operation room) followed by a continuous infusion at 0.5 *μ*g/kg/h until OLV/CO_2_ artificial pneumothorax ended; (2) normal saline group (NS group), with NS administered as a bolus and a continuous infusion with identical volume and rate as the Dex group. A PCIA pump was used for postoperative analgesia in all patients. The PCIA protocol in the NS group was as follows: sufentanil 250 *μ*g + tramadol 500 mg + granisetron 9 mg. Patients in the Dex group received PCIA pump with an additional dose of Dex (200 *μ*g). The PCIA parameters were as follows: total amount of 200 mL, background infusion rate of 2 mL/h, and bolus dose of 0.5 mL, with a lock-out of 15 min. The masked drugs were provided by the nurse who did not take part in other procedures.

### 2.2. Anesthesia Management

Patients received radial artery cannulation in the preanesthesia care unit, and arterial blood gas analysis was conducted before anesthesia (*t*_0_), before OLV/CO_2_ artificial pneumothorax (*t*_1_), 20 min later (*t*_2_), and 20 min after the end of OLV (*t*_3_). After admission to the operation room, pulse oxygen saturation (SPO_2_), electrocardiogram, bi-spectral index (BIS), and regional cerebral oxygen saturation (rSO_2_) were monitored. Anesthesia was induced with midazolam, sufentanil, propofol, and cisatracurium. Sevoflurane and remifentanil were used for maintenance with BIS 40–60. A low tidal volume (5–7 mL/kg) was applied during OLV/CO_2_ artificial pneumothorax.

### 2.3. Neurocognitive Test

All patients were evaluated by the neuropsychological test battery (NTB; i.e., RBANS) [16] before surgery and 7 days after surgery/before discharge by a trained interviewer blinded to the treatment group. RBANS includes 12 subtests: list learning, story memory, figure copy, line orientation, picture naming, semantic fluency, digit span, coding, list recall, list recognition, story recall, and figure recall, which measure immediate memory, language, attention, visuospatial function, and delayed memory.

TICS-m was carried out before surgery and 7 days and 1 month postoperatively. TICS-m consists of 12 items that assess the cognitive function of immediate and delayed memory, orientation, language, calculation, and conceptual knowledge [17]. The total score is 50, and patients with higher scores have better cognitive function. The activities of daily living (ADLs) included instrument ADLs and basic ADLs with 14 items [18]. For each item, scoring from 1 to 4, higher scores, indicate poorer ADLs. The ADLs were assessed at the same time as the TICS-m postoperative test. Pain was evaluated daily in the first three days after surgery, and postoperative pain was graded by visual analogue scale (VAS).

### 2.4. Criteria for POCD Diagnosis

POCD was diagnosed according to the International Study of POCD1 definition [19]. The calculation formula is as follows:

(1) $$\begin{array}{c} Z=\frac{\Delta x-\Delta x_{C}}{SD\left(\Delta x_{C}\right)},\\ Z_{\text{combined}}=\frac{\Sigma Z\;a,b,c,d,etc}{SD\left(\Sigma Z\text{control}\right)}, \end{array}$$

where Δ*x* is the subtracted preoperative score from postoperative score in the operative group, Δ*x*_*C*_ is the subtracted preoperative score from postoperative score in the non-operative group, *ΣZ* is the sum of the *Z* score in RBANS for each individual in the operative group, and *ΣZ*control is the sum of the *Z* score in RBANS in the nonoperative group.

POCD was defined as the *Z* score less than -1.96 in at least two subtests or if the *Z*_combined_ score was -1.96 or less.

### 2.5. Outcome

The primary outcome was the incidence of POCD, assessed with RBANS before surgery and 7 days after surgery/before discharge. The secondary outcomes included the scores of TICS-m before the surgery, 7 days and 30 days after the surgery. The ADL scores at 7 and 30 days after surgery and VAS scores during rest and activity from 1 to 3 days after surgery were also evaluated in this study. Other indicators including arterial blood gas at *t*_0_, *t*_1_, *t*_2_, and *t*_3_; cerebral regional oxygen saturation; duration of the surgery, anesthesia, and OLV/CO_2_ artificial pneumothorax; hospital stay; and hospitalization expenses were analyzed as well.

### 2.6. Statistical Analysis

Sample size was based on a 46% incidence of POCD in thoracic surgery [20], and we assumed that Dex would reduce the incidence of POCD by 50% with *α* = 0.05 and *β* = 0.2. We would require 126 patients to identify the difference with a two-tailed test.

Numerical variables were expressed as the mean ± standard deviation or median (interquartile range). Categorical variables were shown as frequency (percentage). Continuous variables were analyzed using the *t*-test or Mann–Whitney *U* test, as appropriate, and ranked data were analyzed using the Mann–Whitney *U* test. The chi-square or Fisher's exact test was used for categorical variables. Repeated measurement data were analyzed by repeated measurement data analysis of variance. *P* < 0.05 was considered statistically significant. Statistical analysis was performed using SPSS 24.0 (IBM Inc., Chicago, IL, USA).

## 3. Results

**Figure 1:**
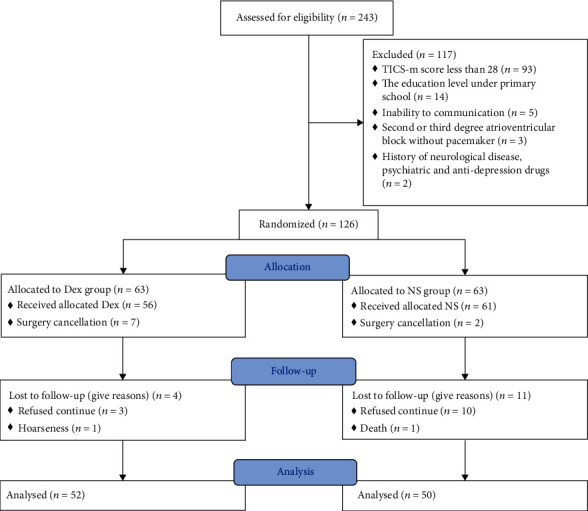
Enrolment flowchart.

A total of 243 patients were screened. Among them, 126 patients were enrolled and randomly allocated to receive either Dex (*n* = 63) or NS (*n* = 63). A total of 52 patients in the Dex group and 50 patients in the NS group completed the RBANS at 7 days postoperatively or before discharge (Figure 1). The demographic and medical data are shown in Table 1. No significant difference was observed in age, gender, and body mass index between the Dex and NS groups. Patients in the Dex group had a shorter hospital length of stay and less expenses than those in the NS group.

### 3.1. Primary Outcome

A total of 35 patients developed POCD at 7 days postoperatively or before discharge, including 19 in the Dex group (36.54%) and 16 in the NS group (32.00%) (*P* = 0.63) (Table 2).

The preoperative and postoperative/before discharge RBANS scores are listed in Table 3. No significant statistical difference was observed between the two groups (*P* > 0.05) (Table 3).

### 3.2. Secondary Outcomes

**Figure 2:**
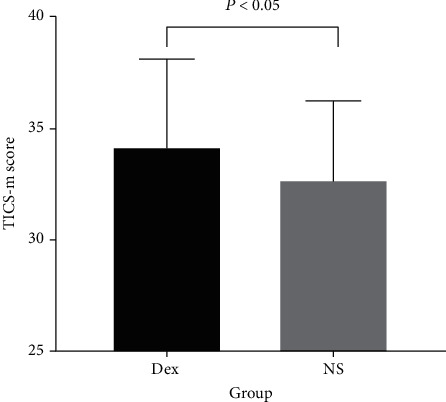
Tics-m score in both groups.

**Figure 3:**
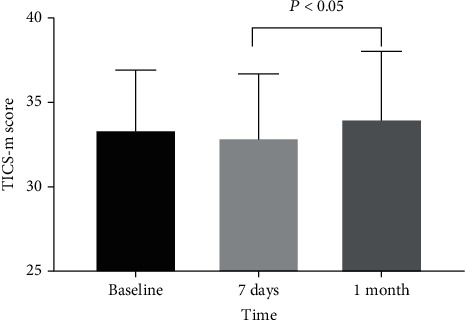
Tics-m score at different times.

The different interventions, Dex or NS, had no influence on the TICS-m score at different times (intervention effect, *P* ≥ 0.05) (Table 4). Compared with those in the NS group, the TICS-m scores were higher in the Dex group (intervention effect, *P* = 0.04) (Table 4) (Figure 2). The TICS-m scores at different times were significantly different (time effect, *P* = 0.02) (Table 4). Compared with that at 7 days postoperatively, the cognitive function improved at 1 month after surgery (Figure 3).

**Figure 4:**
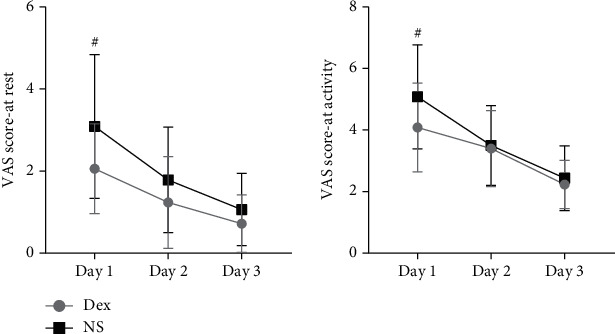
The VAS score after surgery at rest and activity in both groups. ^#^*P* < 0.05, compared with the NS group.

The VAS scores in the Dex group were significantly lower than those in the NS group 1 day postoperation at rest and activity (Figure 4). From the 2nd day after surgery, the VAS scores between the two groups were not statistically different.

**Figure 5:**
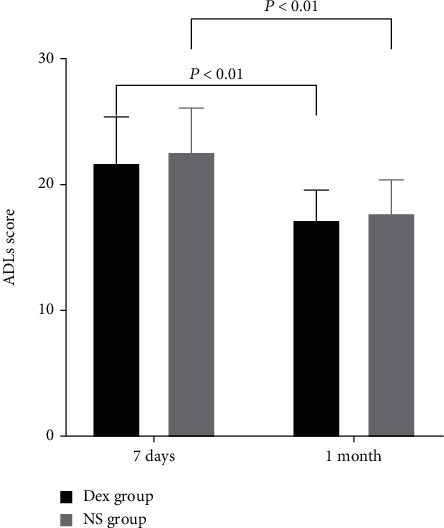
The ADL score in both groups 7 days and 1 month after surgery.

Patients receiving Dex vs. NS had no statistical difference in ADL scores at 7 and 30 days after surgery, but the ADL scores at 30 days after surgery were significantly reduced compared with those at 7 days (Figure 5).

## 4. Discussion

In our study, we found that Dex did not reduce the incidence of early POCD in thoracic surgery. POCD developed in 36.54% (19/52) of patients in the Dex group and 32.00% (16/50) of patients in the NS group. No significant statistical and clinical difference was observed between the two groups. The incidence of POCD 7 days after surgery ranges from 17% to 40% in patients with noncardiac surgery [19, 22, 23]. In thoracic surgery, special ventilation methods induce direct lung injury and systemic inflammation and aggravate postoperative cognitive decline. Egawa et al. found that the incidence of POCD was higher in thoracic surgery, and the incidence was 33.3% and 22.2% in patients undergoing thoracic surgery with sevoflurane and propofol anesthesia, respectively [24].

Our results showed that low-dose Dex in the perioperative period could not improve early cognitive function after thoracic surgery. This finding was in line with a multicenter prospective trial, which was unable to demonstrate a benefit of Dex in POD and POCD and prematurely terminated for futility [15]. In this study, Dex was infused at 0.5 *μ*g/kg/h during surgery and up to 2 h in the recovery room. Some studies [13, 14, 25, 26] hold the view that low-dose Dex could improve cognitive function over a long period. In our study, patients received a loading dose of Dex (0.5 *μ*g/kg intravenously, 15 min after entering the operation room) followed by a continuous infusion at a rate of 0.5 *μ*g/kg/h until OLV or artificial pneumothorax ended. Moreover, the patients in the Dex group received a regular PCIA pump with an additional dose of Dex (200 *μ*g). However, our results showed that Dex could not reduce the incidence of POCD. This may be related to the lower concentration of Dex used in postoperative PCIA. Some studies thought that Dex only suppressed the symptom of agitation in patients undergoing thoracic surgery, rather than improving their cognition [27].

Meanwhile, the incidence of POCD varies with the diagnosis method. The Nomenclature Consensus Working Group recommends that objective cognition decline requires an NTB. So far, there is no unified NTB. As a kind of NTB, RBANS can be used for the repeated measurement of cognitive function, and its validity and test–retest reliability have been determined [28]. Some studies thought that Dex could improve the cognitive decline after surgery by Mini-Mental State Examination (MMSE) and Montreal Cognitive Assessment (MoCA) [29, 30]. In a meta-analysis, Man et al. found that Dex could improve cognitive function, but the subgroup analysis, which included studies using NTB other than MMSE, found that Dex could not improve postoperative cognitive function [31]. Compared with MMSE and MoCA, RBANS is more comprehensive and sensitive to cognitive function. MMSE generally used to screen and has a ceiling effect. MoCA ignores the cultural difference when translating.

In the telephone follow-up, we found that 102 patients had better cognitive function at 1 month after surgery than 7 days after surgery. However, compared with the NS group, Dex could not improve the cognitive function and ADLs at 7 days and 1 month after surgery.

Finally, we found that Dex could relieve the postoperative pain at the first day after thoracic surgery, shorten the hospital stay, and reduce the hospitalization cost. The possible reasons are as follows: (1) postoperative analgesia can promote patients' early activities; enable patients to effectively cough and discharge secretions; and reduce complications such as atelectasis, pneumonia, and deep vein thrombosis. It plays an important role in rapid recovery after thoracic surgery [32]. (2) Dex may reduce opioid requirements, mitigating opioid-related side effects such as nausea and vomiting and respiratory depression [33]. (3) Kim et al. found that Dex reduced emergence agitation in patients undergoing thoracic surgery [27]. It was beneficial to chest drainage. Whether intravenous use of Dex during the perioperative period can promote the early recovery of patients undergoing thoracic surgery requires further clinical research.

This study has several limitations. First, the sample size was small, because many patients could not complete the repeated cognition evaluation, especially the elderly and the less-educated patients. Second, patients were not categorized based on age. The applicability of this conclusion needs further research in elderly thoracic surgery patients. Finally, the long-term cognitive function was not evaluated.

Complex mechanisms and factors are involved in cognitive function. It is difficult to improve patient's cognitive function only by medical treatments. Some studies have shown that multidisciplinary interventions, such as physical exercise; cognitive function training; and improvement of sleep, postoperative anxiety, and depression, may be beneficial for POCD [23, 34, 35].

## 5. Conclusion

Low-dose Dex in the perioperative period did not reduce the incidence of early POCD in thoracic surgery. However, it relieved postoperative pain, decreased hospitalization expenses, and shortened the length of stay.

## Figures and Tables

**Table 1:** Characteristics and intraoperative variables.

	Dex group (*n* = 52)	NS group (*n* = 50)	*P*
Age (year)	59.00 ± 7.72	62.08 ± 7.51	0.08
Sex (male, %)	29 (55.77%)	31 (62.00%)	0.52
BMI	23.83 ± 2.54	23.83 ± 2.54	0.93
Educational level			
Primary	7 (13.46%)	5 (10.00%)	0.71
Junior high school	12 (23.08%)	15 (30.00%)	
Senior high school	14 (26.92%)	12 (24.00%)	
College degree	19 (36.54%)	18 (36.00%)	
TICS-m score (baseline)	34.27 ± 3.79	32.84 ± 2.85	0.14
Smoking history	11 (21.15%)	16 (32.00%)	0.22
Drinking history	5 (9.62%)	9 (18.00%)	0.22
Coronary heart disease	0 (0.00%)	3 (6.00%)	0.11
Hypertension	7 (13.46%)	15 (30.00%)	0.04
Diabetes	2 (3.85%)	7 (14.00%)	0.09
Prolonged rSO_2_ desaturation^∗^	0 [0.00-0.79]	0.02 [0.00-5.12]	0.27
Oxygenation index			
T0	396.34 ± 54.54	409.9 ± 64.71	0.36
T1	396.00 [323.75-494.25]	401.00 [361.00-442.00]	0.86
T2	152.42 ± 69.82	174.30 ± 81.91	0.22
T3	321.26 ± 103.84	314.95 ± 105.87	0.80
Anesthesia duration (min)	174.49 ± 50.18	162.51 ± 42.58	0.27
Surgery duration (min)	106.76 ± 52.07	102.75 ± 34.98	0.69
OLV/artificial pneumothorax duration (min)	80.0 [65.0-99.5]	87.0 [69.0-112.2]	0.44
Length of stay (day)	15.26 ± 3.77	17.69 ± 5.09	0.02
The total hospital cost	52458.71 ± 10649.30	57269.03 ± 9269.98	0.04

BMI: body mass index; TICS-m: Telephone Interview for Cognitive Status-Modified; OLV: one lung ventilation; ^∗^rSO_2_ area under the curve (AUC) of desaturation below 20% of baseline or AUC of desaturation below 50% absolute value [21].

**Table 2:** The incidence of POCD.

	Dex group (*n* = 52)	NS group (*n* = 50)	*P*
Incidence (*n*, %)	19 (36.54%)	16 (32.00%)	0.63

**Table 3:** RBANS scores in both groups.

	Preoperation			Postoperation		
	Dex group	NS group	*P*	Dex group	NS group	*P*
List learning	22.92 ± 4.82	22.38 ± 3.87	0.53	27.65 ± 5.08	27.22 ± 4.84	0.66
Story memory	12.92 ± 4.47	13.08 ± 3.85	0.85	15.54 ± 4.75	16.02 ± 4.24	0.59
Figure copy	15.29 ± 2.84	14.64 ± 3.00	0.27	14.02 ± 2.85	13.78 ± 2.76	0.67
Line orientation	15.65 ± 2.92	14.98 ± 2.87	0.24	15.62 ± 3.11	15.52 ± 2.76	0.87
Picture naming	9.33 ± 1.04	9.14 ± 1.13	0.39	9.42 ± 0.87	9.42 ± 0.86	0.99
Semantic fluency	19.42 ± 3.67	19.14 ± 4.50	0.73	19.35 ± 3.38	18.10 ± 3.82	0.08
Digit span	12.60 ± 2.58	11.98 ± 2.79	0.25	12.52 ± 2.59	11.50 ± 2.79	0.06
Coding	36.83 ± 9.34	33.50 ± 9.70	0.08	35.94 ± 10.44	33.74 ± 10.22	0.29
List recall	3.85 ± 2.35	4.00 ± 2.46	0.75	6.04 ± 2.44	5.34 ± 2.75	0.18
List recognition	18.83 ± 2.12	18.86 ± 1.78	0.93	19.23 ± 1.69	19.24 ± 1.36	0.98
Story recall	6.71 ± 2.23	6.48 ± 2.60	0.63	8.02 ± 2.46	7.34 ± 3.01	0.22
Figure recall	11.79 ± 3.71	11.02 ± 4.19	0.33	12.44 ± 3.58	11.76 ± 4.19	0.38

**Table 4:** The TICS-m scores between both groups.

Group	Time			*P*		
	Preoperative	Postoperative 7 days	Postoperative 1 month	Interactive effect	Time effect	Intervention effect
Dex group	34.05 ± 4.06	33.69 ± 4.11	34.97 ± 3.54	0.74	0.02	0.04
NS group	32.85 ± 2.81	32.26 ± 3.29	33.18 ± 4.29			

## Data Availability

Data for the study can be found in the study. More information concerning the data can be obtained from the corresponding authors.
